# Determinants of Foreign Direct Investment in ASEAN+3 Countries: The Role of Environmental Degradation

**DOI:** 10.3390/ijerph20031720

**Published:** 2023-01-17

**Authors:** Mohd Shahidan Shaari, Muhamad Huzaifah Asbullah, Noorazeela Zainol Abidin, Zulkefly Abdul Karim, Benjamin Nangle

**Affiliations:** 1 Faculty of Business and Communication, Universiti Malaysia Perlis, Kangar 01000, Malaysia; 2 Center for Sustainable and Inclusive Development Studies (SID), Faculty of Economic and Management, Universiti Kebangsaan Malaysia, Bangi 43600, Malaysia; 3 Faculty of Human and Social Sciences, Mykolo Romerio University, LT-08303 Vilnius, Lithuania

**Keywords:** trade openness, infrastructure, corruption, environmental degradation, FDI, panel ARDL, Q01, Q50, L51

## Abstract

Foreign direct investment (FDI) can boost economic growth and provide job opportunities. FDI inflows in ASEAN+3 countries have dropped markedly, which may affect economic development in the region. Many previous studies have investigated a multitude of factors that can influence FDI, such as market size, inflation, trade openness, corruption, and inflation. Previous studies did not, however, consider environmental degradation as a potential factor. Besides corruption and inflation, imposing stringent environmental regulations, such as carbon pricing and taxes to reduce environmental degradation, might deter foreign investors from the country. This is due to heightened costs for foreign investors, which may cause FDI inflows to drop. To shed some light on the reality of this situation, this study examines whether environmental degradation can significantly affect foreign direct investment in the region. This study includes environmental degradation as a potential factor and employs the panel ARDL approach to analyse data from 1995 to 2019. Results show that environmental degradation, infrastructure, and corruption can affect the inflow of FDI in the long run. In the short run, inflation can affect FDI. The findings of this study can be utilized by policymakers in formulating the right policies to attract more investors. An increase in infrastructure facilities should be considered to attract more foreign investment. It is also vital for governments to reduce corruption and inflation to attract more FDI inflows. Environmental incentives should also be introduced to ensure that attempts to reduce environmental degradation do not affect FDI inflows.

## 1. Introduction

Foreign direct investment (FDI) provides international capital to developed and developing countries. Many countries across the globe, especially developing countries, hope to attract more foreign investors to their countries to boost economic activity. Higher economic growth could stem from higher FDI inflows [1]. In the presence of FDI inflows, there will likely be the added benefits of knowledge and technology transfer. In addition, companies can benefit from using new technologies to produce output to meet domestic demand. The relationship between FDI inflows and economic growth remains a topic of debate, though many studies have demonstrated a positive relationship between FDI and economic development [2,3,4,5]. Increased job opportunities can be created with a higher inflow of FDI, and thus more output can be produced, resulting in economic growth. Countries can reap the benefits of having a higher influx of FDI by using more advanced technology and gaining better knowledge. In addition, Ridzuan et al. [6] stated that FDI inflows can also be a potential driver of sustainable development, leading to lower income inequality and improving the quality of the environment.

Due to many previous studies suggesting that FDI can boost economic growth, it is essential to delve into the determinants of FDI. The eclectic theory states that firms invest in another country due to three primary aspects, namely ownership advantages (O), location advantages (L), and internalization (I). As for ownership advantages, firms can control resources (natural or other intangibles such as patents), technology or access to financial capital. Location advantages are likely to arise from lower operating costs and political support from host countries’ governments. As for internalization, firms can produce their products in other countries. The eclectic paradigm suggests that OLI parameters can vary from one firm to another [7,8]. Asiamah et al. [9] and Polyxeni and Theodore [10] explored various determinants of FDI in different countries, such as market size, inflation, infrastructure, trade openness, etc. A larger market size can affect FDI inflows positively. Hence, countries with a large market can attract more investors as they utilize their resources efficiently and thus reap economies of scale [11,12,13]. This can benefit foreign investors by providing lucrative profits. Several studies found that greater trade openness can boost FDI [14,15,16]. As openness intensifies, trade barriers and regulations are reduced to allow more exports and imports, stimulating economic activity and thus ensuring higher FDI inflows. Furthermore, infrastructure also plays an important role in determining FDI, as mentioned by studies such as Nguea [17], Bakar et al. [18] and Rehman et al. [19]. Foreign investors are more likely to invest in countries with strong infrastructure, with poor infrastructure causing foreign investors to incur high costs and earn lower profits, thus leading FDI inflows to decline.

Due to mixed findings on the relationship between corruption and FDI, it remains inconclusive whether corruption can reduce or boost FDI inflows. Canare [20] and Epaphras and Massawe [21] supported the “grabbing hand” theory that higher corruption, which can increase the cost of investment, can lead to lower FDI inflows. However, some argued that higher corruption can result in an increase in FDI inflows due to the ability of foreign investors to avoid rules and regulations [22,23], supporting the “helping hand theory”. Inflation refers to higher prices of goods and services and thus will reduce FDI inflows. Hence, studies such as Mason and Vracheva [24] supported the idea that inflation could reduce FDI as it can lead to higher costs for foreign investors and, thus, lower profits.

Despite a vast array of previous literature on the factors involved in FDI, environmental degradation as a potential factor in FDI inflows has been sparsely explored. Therefore, this study attempts to fill this gap by investigating the impact of environmental degradation on FDI. A high level of CO_2_ emissions in a country may imply that the country has less stringent environmental regulations. Environmental regulations will increase costs for firms to produce output as they may be required to pay higher taxes if they release more CO_2_ emissions, causing FDI inflows to drop. This is one reason why some firms shift their production to countries with less stringent environmental regulations.

Given this backdrop, this paper contributes to policymakers in the ASEAN+3 countries and the FDI literature in the following ways. Firstly, the policymakers in this region can leverage their pull factors, such as infrastructure development, economic openness, and market size, as well as their institutional quality, to attract more FDI. Secondly, since many countries in the region are now moving towards becoming more environmentally friendly, understanding how environmental degradation can affect the inflow of FDI is crucial in strategizing the concept of green investment through new investment aspirations and FDI policies. Thirdly, in comparison with the previous study that focuses only on the ASEAN-5 countries [25], this present study also extends the existing literature on FDI determinants across the ASEAN-6 member countries (Philippines, Malaysia, Singapore, Indonesia, Thailand, and Vietnam), and their three major trading partner countries, namely China, South Korea, and Japan.

This study focuses on the ASEAN+3 countries primarily because of their mutual agreements to address various issues, including trade and the environment. The region accounts for 19.9% of total world FDI inflows. FDI inflows in this region are also continuously rising and contribute significantly to total global FDI inflows. According to the World Bank World Development Indicators [26], the trend in net inflows of FDI as a percentage of GDP in the ASEAN+3 countries remained uncertain over 12 years from 2007 to 2019, thus meriting the investigation of the main factors at play. Among the countries, Singapore contributed the largest share of total FDI inflows. FDI inflows in Japan accounted for the smallest percentage of total FDI inflows in the region. This was likely due to several investment barriers in the country. In 2009, the ASEAN+3 countries experienced marked drops in their FDI inflows due to the financial crisis.

According to the World Bank World Development Indicators [26], China accounts for the most significant share of CO_2_ emissions in the ASEAN+3 countries and the world. In 2019, the country released 11,503.86 Mt of CO_2_ into the atmosphere. The emissions were primarily from burning fossil fuels, especially coal. In China, coal constituted about 58 percent of total energy usage in 2019. As a developed and small country, Singapore released the least CO_2_ emissions at 56.702 Mt. China’s failure to control CO_2_ emissions may be attributed to its environmental regulations and enforcement. The country banned the construction of new coal-fired power plants in 2016, prompting the use of coal to decline. However, when the ban was lifted in 2018, the number of coal-fired power plants rose dramatically. Hence, China remains the largest coal-producing country in the world, indicating that the country has less strict controls on CO_2_ emissions, which can pave the way for higher FDI inflows. Singapore is the lowest contributor to CO_2_ emissions per capita and has higher awareness and strict environmental regulations on CO_2_ emissions. It started to impose a carbon tax of SGD 10–20 on every ton of greenhouse gas produced by firms in 2019. Firms that emit more than 25,000 tons of CO_2_ must pay SGD 5 (USD 3.70) for one ton of emission from 2019 to 2023. After 2023, Singapore plans to improve its strategy to reduce CO_2_ emissions by raising the tax to SGD 10–15. Policies of this kind may be effective in reducing overall CO_2_ emissions to reduce rises in the global temperature, and this policy has resulted in Singapore being the least polluting of the ASEAN+3 countries. This policy may harm FDI as investors are less interested in investing in the country due to additional costs.

The structure of this paper is as follows. A concise assessment of the literature on the correlation between major macroeconomic factors and military spending is provided in Section 2. Section 3 presents the research approach followed for this study. The discussion of the results is presented in Section 4, and the main conclusions and suggestions for policy are covered in Section 5.

## 2. Literature Review

Since FDI can facilitate economic progress, an investigation into its numerous drivers is warranted [27]. A vast array of the previous literature has explored the determinants of FDI across the globe, such as Asbullah et al. [28], Suryanta and Patunru [29], Pečarić et al. [30], etc. Ang [31] focused on Malaysia using an analysis of time-series data from 1960 to 2005. The findings revealed market size measured in GDP can positively impact FDI inflows. In addition, trade openness and infrastructure development can also contribute to higher FDI inflows in the region. Higher corporate taxes and lower exchange rates could reduce FDI inflows. Unexpected results were also found, showing that FDI inflows might increase amid macroeconomic uncertainty. A study conducted by Yohanna [32] that focused on Nigeria, using time-series data from 1981 to 2011, also found that market size, human capital, infrastructure, inflation, and energy consumption can have positive impacts on FDI in the region. Higher exchange rates and interest rates will have negative impacts on FDI. The results were obtained with the employment of the ordinary least squares (OLS) method.

Looking into the determinants of FDI inflows in the pharmaceutical industry, Li et al. [33] embarked on their investigation using an econometric approach and discovered that FDI inflows in the industry are also positively affected by location advantages, such as market size and agglomeration, particularly the pharmaceutical industry output. Information costs and environmental controls can reduce FDI inflows. Similar findings were also obtained by Asongu et al. [34], who investigated the determinants of FDI in the BRICS countries, comprising Brazil, Russia, India, China and South Africa, and also the MINT countries, consisting of Mexico, Indonesia, Nigeria and Turkey. Despite covering many countries with different econometric techniques, the study supported that market size, infrastructure and trade openness can influence FDI. In addition, natural resources and the institutional index do not appear to have any significant impact on FDI in the regions.

Saini and Singhania [35] examined the determinants of FDI in developed and developing countries. Data ranging from 2004 to 2013 with a different technique of analysis, namely the generalised methods of moment (GMM) approach. The results consistently showed that greater trade openness can lead to higher FDI inflows in developing and even developed countries. However, GDP growth and the freedom index can have significant impacts on FDI in developed countries, while gross fixed capital formation and efficiency change measured by productivity growth due to changes in the input-output ratio can significantly affect FDI in developing countries. Employing the same method, Kapuria and Singh [36] conducted their study on South Asia and the West. The results showed that previous FDI inflows might affect current FDI inflows. There are also relationships between corruption, research and development, and the number of trademark applications and FDI.

Differently, Pečarić et al. [30] focused on the determinants of FDI in the services and manufacturing sectors for 10 Central and East European EU countries (CEE), particularly Estonia, Latvia, Lithuania, Slovakia, Slovenia, Bulgaria, Croatia, Hungary, Poland and Romania. Data ranging from 1995 to 2019 were analysed using panel data analysis. The results split into two categories showed that a better credit market and purchasing power might result in higher FDI inflows in the services sector. Better market size and lower exchange rates can contribute to higher FDI inflows in the manufacturing sector. Nguyen and Cieślik [37] focused on FDI from Europe into Asia. Data from 38 European countries and 24 host Asian countries range from 1995 to 2013. The results indicated that income and similar market size spur horizontal FDI growth. Different skilled labour endowments between the two regions can positively impact FDI inflows from Europe to Asia. Furthermore, costs, including trade costs and exchange rate volatility, can reduce FDI inflows.

The most current research on the determinants of FDI, such as Suryanta and Patunru [29], Sookram et al. [38] and Asbullah et al. [28], also supported that market size plays an important role in determining FDI inflows. Asbullah et al. [28] reviewed the previous literature on the determinants of FDI. They concluded that besides market size, infrastructure and trade openness can positively influence FDI. However, inflation can reduce FDI inflows. Corruption remains inconclusive as it may have either a positive or negative impact on FDI. With the employment of the ARDL approach and data ranging from 2000 to 2019, Sookram et al. [38] conducted their study on nine Caribbean countries, particularly the Bahamas, Belize, Barbados, Jamaica, St. Kitts and Nevis, St. Lucia, Trinidad and Tobago, St. Vincent and the Grenadines and Suriname. The results showed that market size, population growth, gross capital formation and natural source rents can positively impact FDI inflows in the long run in the region. Suryanta and Patunru [29] examined the determinants of FDI in Indonesia from 2004 to 2012, considering its 42 trade partners. In contrast to other studies, they included the role of institutional measures, and the results revealed that FDI can be explained by market size, skilled labour and physical labour. Besides, corruption might affect FDI inflows in Indonesia. Trade openness and infrastructure can boost FDI inflows in the region.

Based on the previous literature, several factors can influence FDI, such as market size, inflation, etc. However, environmental degradation as a potential factor has been sparsely investigated. Few studies focus on the relationship running from FDI to environmental degradation. Therefore, this study attempts to address gaps in the literature with a focus on the following aspects. Firstly, this study focuses on the potential conflicts which occur when a significant amount of FDI is invested into an ASEAN+3 country that cannot sufficiently manage environmental regulation and lacks research directly related to the empirical approach. Many studies focus on the importance of FDI regarding environmental degradation, but this study focuses on the impact of environmental degradation on FDI, which is essential to encourage green FDI that incorporates new green technologies that can reduce environmental degradation through investment.

## 3. Research Methodology

### 3.1. Data and Variables Explanation

This study examines the determinants of FDI in the ASEAN+3 countries: the Philippines, Malaysia, Singapore, Indonesia, Thailand, Vietnam, China, South Korea, and Japan. The panel ARDL approach is employed in this study to analyse data on FDI, which is measured as net inflows (% of GDP) [13,15]. According to the eclectic theory, FDI is dependent on three factors, namely ownership advantages, location advantages, and internalisation. This theory serves as a foundation for this study, and thus, market size, inflation, infrastructure, and trade openness are treated as independent variables. It is expected that there is a positive relationship between market size and FDI inflows. Inflation is expected to be negatively associated with FDI, while infrastructure is expected to be positively connected with FDI. Trade openness plays an important role in boosting FDI inflows, and hence, it is expected to have a positive relationship between the two variables. In addition, this study includes another potential determinant of FDI—environmental degradation, which is expected to have a positive connection with FDI.

Inflation is measured as consumer price (annual %) [13]. This data selection is based on the eclectic theory and previous studies. Trade openness is measured as trade (% of GDP) [11,13]. Infrastructure is measured as mobile cellular subscriptions (per 100 people) [27], market size is measured as economic growth [11,39], environmental degradation is measured as CO_2_ emissions, and corruption is measured as the corruption perception index (CPI) [40]. All the data ranging from 1995 to 2019 were extracted from the World Bank World Development Indicators [26]. Table 1 shows the variable descriptions that were used in this study.

### 3.2. Estimation Procedures

#### Panel Unit Root Test

The panel unit root test was carried out before the panel integration test and panel estimation. All the variables were conducted through a panel unit root test. When using panel data, this test is used to guarantee that there is no potential for incorrect regression [41,42]. The panel unit root test is performed primarily to address the low power issue that arises when ADF is used.

Before conducting a panel ARDL test, a panel unit root test must be performed. This panel data analysis uses data from many years (T) and a smaller number of countries (N). Thus, a panel unit root test is crucial to determine the stationarity of data. The unit root tests based on ADF and IPS are carried out as they can be used if heterogeneity exists. Y is the selected variable, i is the country, t is the year, α is the individual fixed effect and ρ is selected to cause the residuals to be uncorrelated over time. After the stationary of the variables is identified, the panel ARDL approach can be used. The panel unit root tests have the following equation:

(1) $$\Delta Y_{\mathrm{it}}=\alpha_{i}+\rho_{i}Y_{i,t-j}+\sum_{i=1}^{\mathrm{pi}}\emptyset_{\mathrm{ij}}\Delta Y_{i,i-j}+\varepsilon_{\mathrm{it}};i=1,2,\ldots N;t=1,2,\ldots .T$$

${\mathrm{FDI}}_{\mathrm{it}}$ represents a foreign direct investment, ${\mathrm{EG}}_{\mathrm{it}}$ represents the market size, ${\mathrm{INFRA}}_{\mathrm{it}}$ represents infrastructure, ${\mathrm{IR}}_{\mathrm{it}}$ represents inflation ${\mathrm{LNCO}}_{2_{\mathrm{it}}}$ represents environmental degradation, ${\mathrm{TRADE}}_{\mathrm{it}}$ represents trade openness, ${\mathrm{COR}}_{\mathrm{it}}$ represents corruption, $\emptyset_{j}$ represents the parameter, $\varepsilon_{\mathrm{it}}$ represents the error term, and $\alpha_{i}$ represents the intercept. The model specification is as follows:

(2) $${\mathrm{FDI}}_{\mathrm{it}}=\alpha_{i\;}+\;\emptyset_{1\;}{\mathrm{EG}}_{\mathrm{it}}\;+\;\emptyset_{2}{\mathrm{INFRA}}_{\mathrm{it}\;}+\emptyset_{3}{\mathrm{IR}}_{\mathrm{it}}\;+\emptyset_{4}{\mathrm{LNCO}}_{2_{\mathrm{it}}}\;+\;\emptyset_{5}{\mathrm{TRADE}}_{\mathrm{it}}\;+\;\emptyset_{6}{\mathrm{COR}}_{\mathrm{it}}\;+\varepsilon_{\mathrm{it}}$$

The panel ARDL approach comprises three main estimators, namely the pooled means group (PMG), means group (MG) and dynamic fixed effects (DFE). These estimators can estimate the long-run and short-run effects of market size, inflation, infrastructure, corruption, environmental degradation, and trade openness on FDI in the ASEAN+3 countries. The generalised method of moments (GMM) method is not the best approach as it is suitable for dynamic micro panel data, such as data across firms. Other than that, the GMM method produces inconsistent coefficients in the long run; thus, it can lead to misleading policy implications. In the PMG estimation, no problem occurs if there is heterogeneity in the short run and homogeneity in the long run. Because the level of heterogeneity is very low, the PMG approach is the most suitable. The vector error correction model (VECM) method is not important, and thus, this study does not employ the method [43]. The panel ARDL approach can be employed even though the variables used in this study are I(0), (I), or both I(0) and I(I). It can also include various lags and can be applied to restricted sample data sizes. The standard function of panel ARDL, which is adapted to this study, is as follows:

(3) $$\begin{array}{ll} \Delta lnFDII_{it}=\alpha + & \overset{k}{\underset{i=1}{\sum }}\beta_{1}lnFDI_{i,t-j}+\overset{1}{\underset{i=0}{\sum }}\beta_{2}lnGDP_{i,t-j}+\overset{m}{\underset{i=0}{\sum }}\beta_{3}\ln COR_{i,t-j}+\overset{n}{\underset{i=1}{\sum }}\beta_{4}\ln TRADE_{i,t-j}+\overset{\circ }{\underset{i=1}{\sum }}\beta_{5}\ln CO2_{i,t-j}\\ & +\overset{p}{\underset{i=1}{\sum }}\beta_{6}lnCPI_{i,t-j}+\overset{q}{\underset{i=1}{\sum }}\beta_{7}\ln INF_{i,t-j}+\partial_{1}lnFDII_{i,t-j}+\partial_{2}\ln GDP_{i,t-j}+\partial_{3}\ln COR_{i,t-j}+\partial_{4}\ln TRADE_{i,t-j}\\ & +\partial_{5}\ln CO2_{i,t-j}+\partial_{6}lnCPI_{i,t-j}+\partial_{6}\ln INF_{i,t-j}+\varepsilon_{it} \end{array}$$

T is the number of years, and N is the number of countries. In Equation (1), i = 1,…..N, t = 1,…..T, Δ is the first variation factor, and ε is the random disturbance term. To apply this method, T must be larger than N, while to use the GMM method, N must be larger than T.

The Hausman test is an econometric statistical test introduced by Hausman [44]. This study conducted the Hausman test to choose either MG or PMG and MG or DFE. If the null hypothesis is rejected, it can be concluded that the MG estimator is more appropriate. If the alternative hypothesis is rejected, it means that the PMG estimator is more efficient.

Besides the PMG, MG and DFE estimators, this study also performed fully modified ordinary least squares (FMOLS) and dynamic ordinary least squares (DOLS) to check the robustness of the results.

## 4. Empirical Results

Descriptive statistics of the data used in this study are reported in Table 2. From the table, it can be learned that FDI shows the highest maximum (26.3963) while market size shows the lowest maximum (2.7425). This study collected data for the period 1995–2019 from the ASEAN+3 countries. Therefore, the number of observations stands at 224. FDI exhibits the largest mean with its value of 21.4044, while inflation exhibits the lowest mean (1.1789).

Before we proceed with our analysis of the determinants of FDI, it is important to check multicollinearity in our regression analysis. Hence, a VIF test is conducted, and the results are reported in Table 3. The values of VIF for all variables are lower than 10. This suggests that there is no multicollinearity; thus, our model can be regressed.

Table 4 shows results from the unit root test by the augmented Dickey–Fuller (ADF) and Im–Pesaran–Shin (IPS) methods. The tests produce results at the level and first difference. The results from the ADF at the level show that all variables (FDI, market size, environmental degradation, trade openness, corruption, and infrastructure) are not significant, except for inflation. This indicates that the null hypothesis in the study is accepted. However, all the variables are significant at the first difference, indicating that all variables are stationary, and thus, the alternative hypothesis is accepted. To prove the accuracy of the ADF, the IPS shows only two variables (market size and inflation) that are significant at the level, but all variables are significant at the first difference.

The next modelling issue that needs to be dealt with is whether there is co-integration. The panel co-integration test will be performed after the panel unit root test. This is to see if there is a long-term relationship between all the factors. Pedroni [45] and Maddala and Wu [46] were the first to apply panel co-integration. Pedroni looked at panel co-integration with heterogeneous intercepts and coefficients, whereas Maddala and Wu focused on combining tests to obtain the test statistic for the entire panel. According to Roudet et al. [47], panel co-integration analysis can improve estimator efficiency.

Table 5 shows the results of panel co-integration. From the table, it can be understood that the results with a trend are mixed, with 3 of 7 statistics rejecting the null hypothesis, while those without a trend are mixed, with 4 of 7 statistics rejecting the null hypothesis. Thus, long-run and short-run relationships can be estimated using the ARDL technique [48]. However, it is necessary to see the error correction term (ECT) to confirm the co-integration. If the value of the ECT is significantly negative and lower than 1, it can be concluded that our variables are co-integrated, which indicated that there is long run comovement between the variables.

Table 6 shows the results of long-run estimation using three estimators, namely PMG, MG and DFE. The Hausman test was used to determine whether the PMG or MG is a better model. The results of the Hausman test show that the *p*-value is 0.9438, and thus the alternative hypothesis can be rejected. This suggests that PMG is the most efficient estimator for our model. The Hausman test was further used to similarly evaluate the PMG and DFE models. The results of PMG show that infrastructure can significantly and positively affect FDI in the long run. This result is consistent with the result of DFE. The coefficient value is 0.5601. This indicates that a 1% increase in infrastructure can increase FDI by 0.5601%. Better developed infrastructure will encourage more investment due to resulting strategic locations. These findings are consistent with the findings of Kumari and Sharma [49] and Jaiblai and Shenai [27]. Aside from this, the results also show that environmental degradation can significantly influence FDI. This implies that a 10% rise in environmental degradation can result in FDI increasing by 2.2865%. Countries with high trade openness facilitate access to their economy to foreign investors; thus, their FDI may escalate.

However, the MG and DFE estimators do not produce any significant results for environmental degradation. Other than that, the results also show a negative relationship between corruption and FDI. This means that if the corruption perception index goes up by 1%, FDI will decrease by −2.2688 %. This means that foreign investors can avoid unwieldy bureaucracy and various regulations to invest in the countries. The results of MG show that corruption does not affect FDI in the long run, but the result of DFE shows a significant positive impact on FDI. This means that if the corruption perception index goes up by 1%, FDI will increase by 20.8230%. The other factors, namely market size, inflation and trade openness, do not influence FDI in the long run.

Table 7 shows the results of short-run estimation using three estimators, namely PMG, MG and DFE. The value of ECT is negatively significant at 1%, indicating the presence of a long-term relationship between variables. The results of PMG indicate that inflation can significantly affect FDI in the short run. This result is not consistent with the results of MG and DFE. This is because if labour costs in the country are high, it is difficult to convince foreign investors to invest in the sector in the ASEAN+3 countries. As a result, the government must be cautious about raising minimum wages, as this could lead to higher labour costs and, as a result, a decrease in total FDI inflows into the ASEAN+3 countries. From that, inappropriate monetary policies may affect inflation in these countries.

Table 8 shows the results of the short-term effects in the ASEAN+3 countries. From the table, only corruption can significantly affect FDI in Indonesia and the Philippines However, the other variables do not show any significance. Thus, the other factors, particularly market size, infrastructure, inflation, environmental degradation, and trade openness, do not affect FDI in the country. Market size is the only variable that seems to affect FDI in Japan. The other factors, particularly infrastructure, environmental degradation, inflation, trade openness, and corruption, do not significantly affect FDI in Japan. Market size, inflation, environmental degradation, and trade openness can significantly affect FDI in Malaysia. The other factors do not have any significant impact on FDI in Malaysia. In Singapore, several factors, such as market size, inflation, and trade openness, show a significant effect on FDI, while the other factors, such as infrastructure, environmental degradation, and corruption, do not affect FDI. Market size, environmental degradation and trade openness can significantly cause FDI to change in South Korea. In Thailand, two factors can affect FDI, namely market size and infrastructure. In Vietnam, variables such as market size and trade openness can affect FDI.

Corruption can reduce FDI inflows in the Philippines and Indonesia, where it has been rampant in the governments of these countries, driving up costs for investors. Market size plays an important role in boosting FDI inflows in Japan, Singapore, South Korea, Malaysia, and Thailand, as these countries have witnessed high GDP growth per capita in comparison with the other ASEAN+3 countries, such as Indonesia, Vietnam, and others. A greater market size in Vietnam may lead to a higher cost of labour that might prompt a reduction in FDI inflows. Inflation can be seen to boost FDI inflows in Singapore and Malaysia, suggesting that foreign investors are not dissuaded from investing in these countries despite their high inflation rates, owing to their good economic performance in the ASEAN. Inflation can lead governments to increase interest rates, which might attract more foreign investors. Infrastructure development seems to reduce FDI inflows only in Thailand. As infrastructure improves in the country, constant maintenance is required. Thus, infrastructure users need to pay for the cost of maintenance, resulting in lower FDI inflows. Environmental degradation can increase FDI inflows in Malaysia as it might provide profits for investors, in line with a less stringent regulatory policy on the environment. However, more stringent environmental regulations in South Korea, with its target of cutting emissions by 40%, have deterred foreign investors from investing in the country.

FMOLS and DOLS tests are conducted to check the robustness of the results, and the results are reported in Table 9. Both show that higher environmental degradation can increase FDI, suggesting that countries with less stringent environmental regulations will attract more foreign investors. Inflation is also found to have a negative impact on FDI from both results. This indicates that higher inflation will increase costs and reduce profits for investors, and hence they are less likely to invest in the countries. The results of both estimators also reveal that infrastructure plays an important role in boosting FDI inflows, implying that poor infrastructure will deter investors from investing in the countries. The results of FMOLS show that there is a negative relationship between market size and FDI inflows.

## 5. Summary and Conclusions

This study aims to investigate the impact of environmental degradation on FDI in the ASEAN+3 countries, namely Thailand, Vietnam, Indonesia, Malaysia, Philippines, Singapore, China, South Korea, and Japan. The panel ARDL approach consists of three estimators (PMG, MG and DFE). The results show that infrastructure and environmental degradation can positively and significantly influence FDI in the long run. Other than that, corruption can have a significant and negative impact on FDI in the long run. The results of each country show that market size can influence FDI in Japan, Malaysia, Singapore, South Korea, Thailand, and Vietnam, while infrastructure factors can influence FDI in Thailand. Inflation can have a short-run impact on FDI in Malaysia and Singapore. Environmental degradation can cause FDI to change in Malaysia and South Korea. Trade openness can influence FDI in Malaysia, Singapore, South Korea, and Vietnam. Lastly, corruption can significantly affect FDI in Indonesia and the Philippines.

These new findings are relevant to policymakers for the ASEAN+3 countries in the following ways. First, policymakers must formulate policies that can boost FDI and thus ensure higher economic growth. The governments should increase spending on infrastructure improvement to boost FDI. Second, governments should remove unwieldy bureaucracy to encourage the inflow of FDI because higher bureaucracy will not attract FDI. Third, since environmental degradation can deter foreign investors from making investments, governments should give environmental incentives such as subsidies to foreign firms. The replacement of non-renewable with renewable energy will not deter foreign investors, and at the same time, sustainable development can be achieved. Although our results do not distinguish whether FDI inflows to countries are green or not, the ASEAN+3 countries need to ensure that in attracting FDI, they enact policies that will subject all FDI inflows to environmental impact assessment. The FDI campaign should emphasize green FDI that focuses on FDI that can encourage +3 countries to internalize the adverse environmental externalities associated with industrial production. Doing so can promote a significant reduction in environmental emissions.

Second, our results also offer policy implications in that the ASEAN+3 countries cannot adopt a one-size-fits-all environmental policy in combating various types of pollutants. From our results, we find that the emission reduction effect of FDI is minimal. CO_2_ is an international pollutant and is poorly controlled locally because its adverse effects are global. Therefore, this CO_2_ requires a mixed strategy in combating different pollutants, especially through cooperative international environmental agreements. This agreement should have a mechanism that can punish countries that do not participate in or violate the agreement.

Third, our findings suggest that FDI affects the reduction of emissions, for which the ASEAN+3 countries can lend credence to the pollution haven hypothesis (PHH). Where this discovery is concerned, the ASEAN+3 countries need to implement stricter environmental policies that will ensure FDI inflows to their countries are environmentally friendly. This may also require shared responsibility between countries to ensure that FDI inflow also meets high environmental standards. Therefore, firms that want to transfer their production activities do not transfer with any technology that is not acceptable in the ASEAN+3 countries.

In summary, the general policy implication of the study is that environmental policies should not be uniform for all countries. Environmental policy must be country- and polluter-specific to solve environmental problems faced by a country. A well-designed environmental policy should reflect the specific needs of a country, considering the country’s level of economic development as well as specific environmental pollutants.

## Figures and Tables

**Table 1:** Variable Description.

Variable Name	Proxy	Unit Measurement	Source
FDI inflows (FDI)	Foreign direct investment, net inflows (% of GDP)	% of GDP	The World Bank World Development Indicators [26]
Market Size (EG)	GDP (constant 2010 US$)	US Dollar	The World Bank World Development Indicators [26]
Infrastructure (INFRA)	Mobile cellular subscriptions (per 100 people)	Per 100 people	The World Bank World Development Indicators [26]
Consumer price index	Consumer price index (2010 = 100)	Index	The World Bank World Development Indicators [26]
Trade Openness (TRADE)	Trade (% of GDP)	Percent	The World Bank World Development Indicators [26]
Environmental Degradation (LNCO_2_)	Total CO_2_ emission	Mt	The World Bank World Development Indicators [26]
Corruption (COR)	Corruption perception index	Index	The World Bank World Development Indicators [26]

**Table 2:** Descriptive Statistics Results.

	Mean	Median	Maximum	Minimum	Std. Dev	Observation
FDI	21.4044	22.9049	26.3963	−22.2384	8.2238	224
Infrastructure	2.6663	2.7741	4.0984	0.0339	1.0410	224
Inflation	1.1789	1.3173	4.0851	−0.9970	0.8481	224
Environmental Degradation	5.7618	5.5322	9.3504	3.5350	1.4651	224
Trade Openness	4.4819	4.4429	6.0806	2.8141	0.7712	224
Market Size	1.5722	1.8414	2.7425	−2.6481	0.9091	224
Corruption	3.7254	3.6109	4.5432	2.8332	0.4404	224

**Table 3:** Variance Inflation Factor (VIF) Results.

Variable	VIF	1/VIF
Trade Openness	2.64	0.3792
Corruption	4.18	0.2395
Environmental Degradation	2.86	0.3495
Inflation	1.70	0.5895
Market Size	1.16	0.8649
Infrastructure	3.54	0.2827
Mean VIF	2.68	

**Table 4:** Panel Unit Root Test Results.

Variable	ADF		IPS	
	Level	1st difference	Level	1st difference
FDI	17.3389 (0.4999)	73.2331 *** (0.0000)	−0.12947 (0.4485)	−6.1358 *** (0.0000)
Market Size	85.0575 (0.0000)	151.710 *** (0.0000)	−6.1453 *** (0.0000)	−12.5246 *** (0.0000)
Inflation	44.1905 *** (0.0005)	146.901 *** (0.0000)	−3.7506 *** (0.0001)	−12.1441 *** (0.0000)
Environmental Degradation	9.83526 (0.9372)	81.6904 *** (0.0000)	2.42486 (0.9923)	−6.8700 *** (0.0000)
Trade Openness	14.0155 (0.7281)	90.9859 *** (0.0000)	0.5623 (0.7130)	−7.7137 *** (0.0000)
Corruption	18.9470 (0.3951)	110.166 *** (0.0000)	0.00503 (0.5020)	−9.27139 *** (0.0000)
Infrastructure	28.3173 (0.0574)	47.4135 *** (0.0002)	−1.5776 (0.0573)	−3.5386 *** (0.0002)

Note: *** indicate significance level of 1%. The probability values are in parentheses.

**Table 5:** Panel Co-integration Results.

	Within Dimension	
	Without Trend	With Trend
Panel v-Statistic	−1.293590 (0.9021)	−2.480875 (0.9934)
Panel rho-Statistic	−2.1826 ** (0.0145)	0.0221 (0.5088)
Panel PP-Statistic	−10.0160 *** (0.0000)	−12.2817 *** (0.0000)
Panel ADF-Statistic	0.6231 (0.7334)	0.1014 (0.5404)
	Between Dimension	
	Without Trend	With Trend
Group rho-Statistic	1.4376 (0.9247)	2.7089 (0.9966)
Group PP-Statistic	−10.0449 *** (0.0000)	−15.0728 *** (0.0000)
Group ADF-Statistic	−0.371669 (0.3551)	−1.1883 (0.1174)

Note: *** and ** indicate significance levels of 1% and 5%, respectively. The probability values are in parentheses.

**Table 6:** Long-Run Estimation Results (ARDL).

Variables	PMG	MG	DFE
Market Size	−0.11808 (0.173)	1.7520 (0.430)	2.3920 ** (0.038)
Infrastructure	0.5601 * (0.078)	5.3951 (0.236)	5.3005 *** (0.002)
Inflation	0.0697 (0.440)	2.9341 (0.503)	0.6384 (0.598)
Environmental Degradation	2.2865 *** (0.000)	6.5565 (0.467)	−2.1154 (0.417)
Trade Openness	0.2641 (0.502)	8.7428 (0.319)	−7.2172 ** (0.028)
Corruption	−2.2688 *** (0.001)	4.4094 (0.776)	20.8230 *** (0.000)
Hausman (prob.)	1.72 (0.9438)		25.59 (0.0003)

Note: ***, ** and * indicate significance levels of 1%, 5% and 10%, respectively.

**Table 7:** Short-Run Estimation Results.

Variables	PMG	MG	DFE
ECT	−0.5957 *** (0.000)	−1.0659 *** (0.000)	−0.8331 *** (0.000)
Market Size	1.5460 (0.141)	−0.0700 (0.966)	−0.3100 (0.629)
Infrastructure	−10.0171 (0.308)	−9.3699 (0.430)	2.8024 (0.444)
Inflation	−1.2400 * (0.081)	−0.7751 (0.799)	−0.1858 (0.847)
Environmental Degradation	−31.5081 (0.172)	−48.5712 (0.225)	−18.1093 (0.139)
Trade Openness	−10.6080 (0.383)	−4.0106 (0.733)	−5.6234 (0.370)
Corruption	7.2916 (0.172)	11.1804 (0.275)	−4.3358 (0.479)
Constant	8.5918 *** (0.000)	−68.8432 (0.223)	−24.6926 (0.198)

Note: *** and * show significance levels of 1% and 10%, respectively. The value in the parentheses is the probability value.

**Table 8:** Short-run country-specific results.

Countries	Market Size	Infra.	Inflation	Environmental Degradation	Trade Openness	Corrupt.
China	0.1045 (0.795)	0.5617 (0.340)	4.4015 (0.618)	−1.3367 (0.382)	1.0985 (0.197)	0.7714 (0.204)
Indonesia	6.3103 (0.210)	0.3370 (0.974)	5.4566 (0.395)	−87.7647 (0.245)	8.5375 (0.745)	49.9034 ** (0.030)
Japan	7.7232 ** (0.008)	−88.5742 (0.116)	4.4016 (0.618)	−198.6152 (0.087)	−107.2983 (0.121)	3.8908 (0.923)
Malaysia	0.3450 *** (0.001)	1.3988 (0.403)	0.7935 *** (0.001)	7.2564 ** (0.015)	3.9641 * (0.099)	3.1784 (0.156)
Philippines	−0.1360 (0.371)	1.0484 (0.349)	−0.0657 (0.770)	1.7737 (0.456)	−1.7876 (0.251)	1.6182 ** (0.026)
Singapore	0.1704 *** (0.011)	−4.3149 (0.260)	0.5309 *** (0.005)	0.0732 (0.975)	−5.4146 *** (0.003)	3.6297 (0.326)
South Korea	0.2885 *** (0.005)	0.1321 (0.912)	0.1868 (0.443)	−3.9147 ** (0.036)	1.6660 ** (0.035)	1.4292 (0.168)
Thailand	0.2554 *** (0.000)	−0.8412 * (0.055)	−0.0633 (0.583)	−1.7727 (0.462)	0.2185 (0.872)	0.8251 (0.489)
Vietnam	−1.1470 ** (0.035)	0.0977 (0.616)	−0.0885 (0.151)	0.7285 (0.385)	3.5439 *** (0.000)	0.3786 (0.445)

Note: ***, ** and * show the significance levels of 1%, 5% and 10%, respectively. The probability values are in parentheses.

**Table 9:** Short-run country-specific results.

	FMOLS		DOLS	
Variable	Coefficient	Prob.	Coefficient	Prob.
Environmental Degradation	0.0751 **	0.0238	0.0659 ***	0.0021
Corruption	−0.0183	0.7480	−0.0089	0.6794
Inflation	−0.0167	0.3510	0.0064 ***	0.0026
Market size	−0.0232 **	0.0130	−0.0207	0.2318
Infrastructure	0.9847 ***	0.0000	1.0000 ***	0.0000
Trade openness	−0.0121	0.1995	0.0078	0.5859

Note: *** and ** show significance levels of 1% and 5%, respectively.

## Data Availability

The data presented in this study are available on the World Development Indicators and coun-tryeconomy.com (accessed on 24 June 2022).
